# A propensity-matched study of the association between optimal folic acid supplementation and birth defects in Shaanxi province, Northwestern China

**DOI:** 10.1038/s41598-019-41584-5

**Published:** 2019-03-27

**Authors:** Pengfei Qu, Shanshan Li, Danmeng Liu, Fangliang Lei, Lingxia Zeng, Duolao Wang, Hong Yan, Wenhao Shi, Juanzi Shi, Shaonong Dang

**Affiliations:** 1 Assisted Reproduction Center, Northwest Women’s and Children’s Hospital of Xi’an Jiaotong University Health Science Center, No. 73 Houzaimen, Xi’an, Shaanxi 710003 People’s Republic of China; 2 Translational Medicine Center, Northwest Women’s and Children’s Hospital of Xi’an Jiaotong University Health Science Center, No.1616 Yanxiang Road, Xi’an, Shaanxi 710061 People’s Republic of China; 3 Department of Epidemiology and Health Statistics, School of Public Health, Xi’an Jiaotong University Health Science Center, No. 76 Yanta West Road, Xi’an, Shaanxi 710061 People’s Republic of China; 4 Department of Clinical Sciences, Liverpool School of Tropical Medicine Pembroke Place, Liverpool, United Kingdom

## Abstract

The association between folic acid supplementation and birth defects other than neural tube defects remains unclear. We utilized data from a large population-based survey to examine the association between folic acid supplementation and birth defects in Northwestern China. A total of 29,204 women with infants born between 2010 and 2013 were surveyed in Shaanxi province, Northwestern China, using a stratified multistage sampling method. Propensity scores were used to match 9,293 women with optimal folic acid supplementation with 9,293 women with nonoptimal folic acid supplementation, and the effects of optimal folic acid supplementation on birth defects were assessed by a conditional logistic regression model. After propensity score matching, the overall birth defect rate, cardiovascular system defect rate and nervous system defect rate for the women with optimal folic acid supplementation were lower than those for the women with nonoptimal folic acid supplementation (overall birth defects: OR = 0.71, 95% CI = 0.57–0.89, P = 0.003; cardiovascular system defects: OR = 0.65, 95% CI = 0.44–0.96, P = 0.032; nervous system defects: OR = 0.13, 95% CI = 0.02–0.99, P = 0.049). Optimal folic acid supplementation was associated with a decreased prevalence of birth defects, especially in the cardiovascular system and nervous system. Our findings have important implications for birth defect intervention with folic acid supplementation for countries with a high prevalence of birth defects, such as China.

## Introduction

“Birth defect” is a general term for functional or structural abnormalities in a developing fetus. Some birth defects are caused by known genetic factors, such as chromosome aberrations or genetic mutations, environmental factors, or interactions between genetic factors and environmental factors. Birth defects are the leading cause of early miscarriage, stillbirth, perinatal death, and infant and child death^1^. Approximately 5.6% of pregnancies in China, including both live births and pregnancy losses, are affected by birth defects^2^. There are approximately 0.25 million births with major defects every year in China, which leads to a substantial disease burden^2^.

Both randomized controlled studies and observational studies have confirmed that periconceptional folic acid supplementation can reduce the prevalence of neural tube defects^3–6^. The WHO recommends that all women should take a folic acid supplement from the period before conception until 12 weeks of gestation^7^. In line with the WHO recommendation, China has issued a policy of folic acid supplementation (folic acid supplement ≥400 µg/d) during the 3 months before pregnancy and the first trimester of pregnancy. All Chinese women of childbearing age have access to free folic acid. The policy has played a very important role in significantly preventing neural tube defects (NTDs) in China^2^. However, the relationships between periconceptional folic acid supplementation and other birth defects, such as cardiovascular system defects, oral clefts, and urinary system defects, remain inconclusive. Interpretations of findings from observational studies are often limited by potential residual biases from measured confounders and possible biases resulting from unmeasured confounders. The propensity score (PS) technique has emerged as an effective tool to address selection and residual biases, and PS methods allow the transparent design and analysis of observational studies^8–10^. The objective of this study was to investigate the association between periconceptional folic acid supplementation and birth defects using PS methods in Shaanxi province, Northwest China.

## Results

### Participant characteristics

Among the birth defects surveyed, we identified 192.44 cases (per 10,000 infants) of overall birth defects before PS matching, including 6.85 cases (per 10,000 infants) of nervous system defects; 23.97 cases (per 10,000 infants) of eye, ear, face and neck defects;, 63.35 cases (per 10,000 infants) of cardiovascular system defects; 3.77 cases (per 10,000 infants) of respiratory system defects; 11.64 cases (per 10,000 infants) of oral clefts; 8.56 cases (per 10,000 infants) of digestive system defects; 5.82 cases (per 10,000 infants) of genital organ defects; 2.05 cases (per 10,000 infants) of urinary system defects; 34.58 cases (per 10,000 infants) of musculoskeletal system defects; 0.68 cases (per 10,000 infants) of chromosomal abnormalities; and 31.16 cases (per 10,000 infants) of other defects (Table 1).

**Table 1:** Prevalence of birth defects in infants (per 10,000 infants).

	Nonoptimal folic acid supplementation n = 18,611	Optimal folic acid supplementation n = 10,593	Total n = 29,204
Nervous system	18 (9.67)	2 (1.89)	20 (6.85)
Eye, ear, face and neck	51 (27.40)	19 (17.94)	70 (23.97)
Cardiovascular system defect	131 (70.39)	54 (50.98)	185 (63.35)
Ventricular septal defect	42 (22.57)	15 (14.16)	57 (19.52)
Atrial septal defect	29 (15.58)	14 (13.22)	43 (14.72)
Patent ductus arteriosus	26 (13.97)	7 (6.61)	33 (11.30)
Pulmonary artery constriction	10 (5.37)	5 (4.72)	15 (5.14)
Atrioventricular septal defect	6 (3.22)	4 (3.78)	10 (3.42)
Tetralogy of Fallot	5 (2.69)	3 (2.83)	8 (2.74)
Others	13 (6.99)	6 (5.66)	19 (6.51)
Respiratory system	8 (4.30)	3 (2.83)	11 (3.77)
Oral clefts	28 (15.04)	6 (5.66)	34 (11.64)
Digestive system	15 (8.06)	10 (9.44)	25 (8.56)
Genital organs	10 (5.37)	7 (6.61)	17 (5.82)
Urinary system	4 (2.15)	2 (1.89)	6 (2.05)
Musculoskeletal system	72 (38.69)	29 (27.38)	101 (34.58)
Other defects	64 (34.39)	27 (25.49)	91 (31.16)
Chromosomal abnormalities	1 (0.54)	1 (0.94)	2 (0.68)
Total birth defects	402 (216.00)	160 (151.04)	562 (192.44)

**Figure 1:**
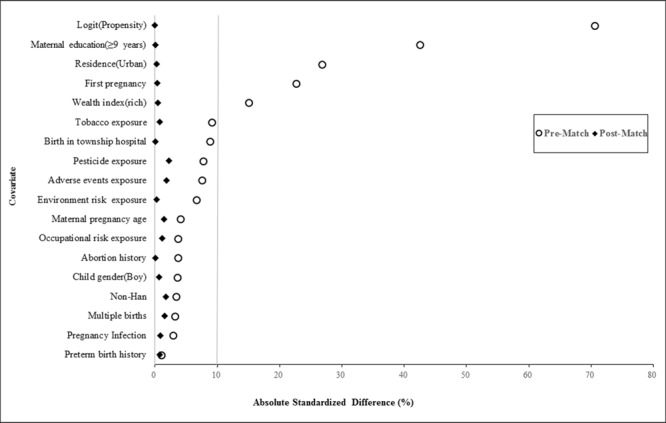
Absolute standardized differences before and after propensity scores matching comparing covariate values for participants with optimal folic acid supplementation and nonoptimal folic acid supplementation.

Among the matched participants, 20.79% were ≥30 years, 46.38% had ≥9 years of education, 0.43% were non-Han, and 35.95% were urban residents. Table 2 compares the participants’ baseline characteristics according to folic acid supplementation before and after PS matching. There were significant prematch imbalances in several important covariates, including multiple births and tobacco exposure, that were balanced after matching. Our PS matching reduced the standardized differences for all covariates below 10% in absolute value, demonstrating substantial improvement in covariate balance across the folic acid supplementation groups (Fig. 1).

**Table 2:** Baseline participant characteristics by folic acid supplement before and after propensity score (PS) matching (%).

	Before PS match			After PS match		
	Nonoptimal folic acid supplementation n = 18,611	Optimal folic acid supplementation n = 10,593	P value	Nonoptimal folic acid supplementation n = 9,293	Optimal folic acid supplementation n = 9,293	P value
Child sex (boys)	10319 (55.45)	5684 (63.66)	0.003	5067 (54.52)	5038 (54.21)	0.669
Multiple births	201 (1.08)	151 (1.43)	0.009	71 (0.76)	84 (0.90)	0.294
Pregnancy age ≥30 years	4510 (24.23)	2385 (22.51)	0.001	1904 (20.49)	1960 (21.09)	0.311
Maternal education (≥9 years)	5732 (30.80)	5429 (51.25)	<0.001	4311 (46.39)	4310 (46.39)	0.988
Non-Han	100 (0.53)	87 (0.82)	0.003	34 (0.37)	45 (0.48)	0.215
Residence (urban)	5245 (28.18)	4322 (40.80)	<0.001	3334 (35.88)	3347 (36.01)	0.842
Wealth index (rich)	8797 (47.27)	5801 (54.76)	<0.001	4918 (52.92)	4940 (53.16)	0.746
First pregnancy	8755 (47.04)	6171 (58.26)	<0.001	5314 (57.18)	5296 (56.99)	0.790
Preterm birth history	549 (2.95)	267 (2.52)	0.032	167 (1.80)	177(1.90)	0.586
Abortion history	2500 (13.43)	1557 (14.70)	0.003	1254 (13.49)	1255 (13.50)	0.983
Pregnancy infection	2370 (12.73)	1451 (13.70)	0.019	1111 (11.96)	1136 (12.22)	0.574
Birth at a township hospital	2519 (13.54)	1129 (10.66)	<0.001	986 (10.61)	983 (10.58)	0.943
Pesticide exposure	269 (1.45)	70 (0.66)	<0.001	37 (0.40)	51 (0.55)	0.135
Tobacco exposure	11366 (61.07)	5993 (56.58)	<0.001	5433 (58.46)	5398 (58.09)	0.603
Adverse events exposure	1374 (7.38)	587 (5.54)	<0.001	424 (4.56)	460 (4.95)	0.215
Occupational risk exposure	1607 (8.63)	807 (7.62)	0.002	610 (6.56)	637 (6.85)	0.429
Environment risk exposure	5145 (27.64)	2621 (24.74)	<0.001	2233 (24.03)	2243 (24.14)	0.864

### Optimal folic acid supplementation and birth defects

After PS matching, there were 195.85 cases per 10,000 infants in the nonoptimal folic acid supplementation group and 138.89 cases per 10,000 infants in the optimal folic acid supplementation group (OR = 0.71; 95% CI = 0.57–0.89; *P* = 0.049). Compared with 8.61 nervous system defects per 10,000 infants in the nonoptimal folic acid supplementation group, there were 1.08 nervous system defects per 10,000 infants in the optimal folic acid supplementation group (OR = 0.13; 95% CI = 0.02–0.99; *P* = 0.049). Compared with 67.79 cardiovascular system defects per 10,000 infants in the nonoptimal folic acid supplementation group, there were 44.12 cardiovascular system defects per 10,000 infants in the optimal folic acid supplementation group (OR = 0.65; 95% CI = 0.44–0.96; *P* = 0.032) (Table 3). Among cardiovascular system defects, the pregnancy outcomes of the optimal folic acid supplementation group were less likely to involve ventricular septal defects, atrial septal defects and patent ductus arteriosus than those of the nonoptimal folic acid supplementation group (ventricular septal defect: OR = 0.58, 95% CI = 0.30–1.13, *P* = 0.109; atrial septal defect: OR = 0.83; 95% CI = 0.36–1.93, *P* = 0.672; patent ductus arteriosus: OR = 0.43, 95% CI = 0.17–1.12, *P* = 0.083). However, the relationships between ventricular septal defects, atrial septal defects, patent ductus arteriosus, pulmonary artery constriction, atrioventricular septal defects and tetralogy of fallot and folic acid supplementation were not statistically significant.

**Table 3:** OR and 95% CI for birth defects by optimal folic acid supplement in conditional logistic regression.

	OR (95% CI)	P value
Nervous system	**0.13 (0.02–0.99)**	**0.049**
Eye, ear, face and neck	0.50 (0.25–1.01)	0.055
Cardiovascular system defect	**0.65 (0.44–0.96)**	**0.032**
Ventricular septal defect	0.58 (0.30–1.13)	0.109
Atrial septal defect	0.83 (0.36–1.93)	0.672
Patent ductus arteriosus	0.43 (0.17–1.12)	0.083
Pulmonary artery constriction	1.00 (0.25–4.00)	0.999
Atrioventricular septal defect	1.00 (0.20–4.96)	0.999
Tetralogy of Fallot	1.50 (0.24–9.00)	0.658
Respiratory system	0.75 (0.17–3.35)	0.706
Oral clefts	0.46 (0.18–1.21)	0.117
Digestive system	0.67 (0.32–2.41)	0.796
Genital organs	1.50 (0.42–5.32)	0.530
Urinary system	1.00 (0.06–15.99)	0.999
Musculoskeletal system	1.00 (0.57–1.76)	0.999
Other defects	0.93 (0.55–1.58)	0.785
Chromosomal abnormalities	1.00 (0.06–15.99)	0.999
Total birth defects	**0.71 (0.57–0.89)**	**0.003**

After PS matching, the pregnancy outcomes of the optimal folic acid supplementation group were less likely to include eye, ear, face and neck defects; respiratory system defects; oral clefts and digestive system defects than those of the nonoptimal folic acid supplementation group (eye, ear, face and neck defects: OR = 0.50, 95% CI = 0.25–1.01, *P* = 0.055; respiratory system: OR = 0.75, 95% CI = 0.17–3.35, *P* = 0.706; oral clefts: OR = 0.46, 95% CI = 0.18–1.21, *P* = 0.117; digestive system: OR = 0.67, 95% CI = 0.32–2.41, *P* = 0.796). However, the relationships between folic acid supplementation and respiratory system defects, oral clefts, digestive system defects, abnormalities of the genital organs, urinary system defects, musculoskeletal system defects, chromosomal abnormalities and other defects were not statistically significant.

### Sensitivity analyses

In the full (prematched) group of participants (n = 29204), there were 216.00 cases of birth defects per 10,000 in the nonoptimal folic acid supplementation group, compared with 151.04 cases of birth defects per 10,000 in the optimal folic acid supplementation group (OR = 0.70; 95% CI = 0.58–0.84; *P* < 0.001). Compared with 9.67 cases of nervous system defects per 10,000 in the nonoptimal folic acid supplementation group, there were 1.89 cases of nervous system defects per 10,000 in the optimal folic acid supplementation group (OR = 0.20; 95% CI = 0.5–0.84; *P* = 0.028). Compared with 70.39 cases of cardiovascular system defects per 10,000 in the nonoptimal folic acid supplementation group, there were 50.98 cases of cardiovascular system defects per 10,000 in the optimal folic acid supplementation group (OR = 0.72; 95% CI = 0.53–0.99; *P* = 0.045). When we adjusted for PS, the association remained significant (birth defects: OR = 0.70, 95% CI = 0.58–0.89, *P* = 0.003; nervous system defects: OR = 0.20, 95% CI = 0.05–0.84, *P* = 0.030; cardiovascular system defects: OR = 0.72, 95% CI = 0.45–0.99, *P* = 0.032). There were no significant differences in other defects between the folic acid supplementation groups.

Among the participants in PS tertiles two and three (n = 19936), we observed similar effects on optimal folic acid supplementation on birth defects, nervous system defects and cardiovascular system defects: PS-adjusted (birth defects: OR = 0.79, 95% CI = 0.65.−0.95, *P* = 0.013; nervous system defects: OR = 0.21, 95% CI = 0.05–0.87, *P* = 0.031; cardiovascular system defects: OR = 0.72, 95% CI = 0.52–0.99, *P* = 0.047).

## Discussion

Shaanxi province is a high-prevalence area of birth defects in Northwest China. The overall prevalence of birth defects and cardiovascular system defects in Shaanxi province is higher than the national average and the averages for other provinces in Northwest China^2^. Thus, the prevention of birth defects and improvement of population health in Shaanxi province, Northwest China, are urgently necessary. This study investigated the association between folic acid supplementation and the birth defect spectrum, and our findings demonstrated that optimal folic acid supplementation was associated with a decreased risk of overall birth defects in Northwest China, especially nervous system and cardiovascular system defects. Optimal folic acid supplementation was not found to be associated with a significant decrease in other defects. These findings are very important because of the high prevalence of birth defects and the inconsistent rate of periconceptional folic acid supplementation in Northwest China.

Nervous system defects, especially NTDs, are common congenital anomalies in China. Nervous system defects always occur very early in pregnancy. The prevalence of NTDs was 13.8 per 1,000 births in 1999 in northern China, compared with less than 1 per 1,000 births in the USA^11,12^. A China-U.S. collaborative project for neural tube defect prevention conducted in northern and southern China revealed an 85% reduction in NTD risk in the northern region and a 40% reduction in NTD risk in the southern region^6^. Consequently, folic acid supplementation had a much greater effect on reducing the occurrence of NTDs in China. In our study, a lower prevalence of 6.85 per 10,000 infants was also observed. A large number of clinical trials and observational studies have shown that folic acid supplementation during pregnancy reduced the prevalence and recurrence rate of NTDs^13–16^, and an ecological study found that the prevalence of anencephaly and spina bifida decreased to 11% from 20% and to 21% from 34%, respectively, after folic acid fortification^17^. Our study was based on a birth defects survey in folate deficient areas in Northwestern China, and focused on the association between folic acid supplementation and birth defects. Although folic acid supplementation prevents NTDs, the dosage of folic acid supplementation and the duration of folic acid supplementation in practice are not consistent among different countries and regions of the world^7^, which are still of great impact on the prevention of NTDs. Our study provides additional evidence that optimal folic acid supplementation during the periconceptional period is associated with a reduction in the prevalence of nervous system defects (OR = 0.13; 95% CI = 0.02–0.99; P = 0.049). Therefore, our study further added to support for the reduction of nervous system defects by means of folic acid supplementation during the periconceptional period.

Cardiovascular system defects rank first among birth defects in China. The prevalence of cardiovascular system defects was 10~20 per 1,000 during the fetal period and 8~10 per 1,000 in live births^18^. From 2000 to 2011, the overall birth defect prevalence rates showed a downward trend. The National Stocktaking Report on Birth Defect Prevention (2012) (China) reported that the prevalence rate of cardiovascular system defects increased between 2000 and 2011^2^. Some studies have confirmed that the use of vitamin supplements containing folic acid in early pregnancy was associated a reduced risk of cardiovascular system defects in offspring. Ionescuittu compared the prevalence of severe congenital heart disease before and after folic acid fortification in Quebec province, Canada^19^. The results showed that there was a significant 6% decrease per year (OR = 0.94; 95% CI = 0.90–0.97) in the seven years after folic acid fortification began. Liu carried out a population-based cohort study of all live births and stillbirths in Canada and found that folic acid fortification was associated with lower rates of nonchromosomal heart defects (OR = 0.89; 95% CI = 0.82–0.98)^20^. A 1984–1991 Hungarian randomized controlled trial (RCT) of periconceptional multivitamin supplementation containing folic acid (0.8 mg) showed a significant reduction in the first occurrence of cardiovascular abnormalities (OR = 0.60; 95% CI = 0.38–0.96), especially ventricular septal defects (OR = 0.26; 95% CI = 0.09–0.72)^21^. However, two case-control studies did not find a reduced risk of cardiovascular system defects as a result of folic acid supplementation^22,23^. In our study, after balancing all measured baseline covariates by PS matching, optimal folic acid supplementation may have reduced the risk of cardiovascular system defects by as much as 35% compared with the nonoptimal folic acid supplementation group. This finding implies the possibility of preventing cardiovascular system defects with folic acid supplementation in maternal and child health care practice during the periconceptional period. The association was analyzed further between the optimal folic acid supplementation and subtype of cardiovascular system defects. Because of lower prevalence, the association between the optimal folic acid supplementation and subtype of cardiovascular system defects and other birth defects were not significant statistically, but those results still were important clues for future study.

Two meta-analyses indicated that multivitamin supplementation with folic acid protected against oral clefts^24,25^. However, there is no strong evidence of an association between oral clefts and folic acid intake alone^25^. Some studies investigated the association between folic acid supplementation and the risk of eye, ear, face and neck defects, urinary system defects, musculoskeletal system defects and chromosomal abnormalities, but the results were inconsistent. Using the American National Birth Defects Prevention Network, Canfield investigated the change in the prevalence of urinary system defects after folic acid fortification and confirmed a reduction in the birth prevalence of renal agenesis, upper limb reduction defects, and omphalocele but an increase in obstructive genitourinary defects and Down syndrome^17^. Some studies have reported that multivitamin supplementation was associated with a reduced risk of limb reduction defects, while the associations were inconsistent for different limb reduction defect types^17,26^. A case-control study showed that a significant protective effect against Down’s syndrome was observed with large doses of folic acid (approximately 6 mg/d) during the first gestational month (OR = 0.4, 95% CI: 0.2 to 0.7)^27^. Unfortunately, these results were inconsistent among studies.

In our study, the prevalence of oral cleft, eye, ear, face and neck defects, respiratory system defects, digestive system defects and some specific birth defects (congenital hydrocephalus, ventricular septal defect, atrial septal defect and patent ductus arteriosus) in the optimal folic acid supplementation group was lower than in the nonoptimal folic acid supplementation group. However, there was no significant difference between the two groups. The lower prevalence of those specific defects could account for this lack of significance to some extent, and future studies with larger samples are required to further investigate these associations.

Previous observational studies usually used traditional regression methods to analyze the association between folic acid supplementation and birth defects^20–23^. However, selection bias and an imbalance of important variables between the groups were major problems in those observational studies^28^. Adjustment by specific covariates in regression model is adopted commonly but number of covariate, collinearity and sample size could influence the efficiency. The PS is a function of multiple covariates and represents the combined action of multiple covariates^29^. For an observational study, PS matching was effective in balancing confounding factors in a similar randomized treatment and reduced the selection bias^8–10^. When there were many covariates entering the model and relatively few events, such as the 17 covariates and 562 cases of birth defects (192.44 cases per 10,000 participants) in our study, PS matching was appropriate for providing an accurate estimated value compared with conventional multivariable methods^30^. Thus, the major strength of this study was the use of PS matching, which balanced optimal folic acid supplementation and nonoptimal folic acid groups on a large number of covariates by using a linear combination of covariates for a single score. To some extent, PS matching also reduced the confounding that may be present in our study. After matching, we further verified the consistency of the association between folic acid supplementation and birth defects by means of 3 models (including model 1, with no adjustment; model 2, with adjusted PS; and model 3, with adjusted covariates and PS). Our sample came from 10 urban districts and 20 rural counties in the province that were randomly selected according to the imbalanced population distribution and fertility level between rural and urban areas, and a stratified multistage sampling method was employed. Thus, our samples had relatively good representativeness and were suitable for evaluating the association between folic acid supplementation and birth defects in the population.

This study had some limitations. First, the recall bias existed possibly in this study because both the folic acid supplementation and risk factor information was obtained through the participant’s recall. Additionally, although we used the PS matching technique to balance selection bias between the two groups, the finding of our study might be potentially confounded by the biases related to unmeasured or hidden covariates because the covariates used for PS matching were limited, resulting in incomplete or inexact matching. In our study, it is unlikely that a hidden variable could be completely unrelated to any of the 17 covariates used in our PS analysis. Second, although the policy of folic acid supplementation has promoted universal periconception folate supplementation in China, the nonoptimal folic acid supplement group still may have been heterogeneous in that it included the women who used no or insufficient folic acid. Even though we tried our best to reduce the misclassification of birth defect diagnoses in our study by including a team of experts in the diagnosis of birth defects, a risk of misclassification of birth defect diagnoses still existed in the cases of deaths with birth defects. Finally, although our study included more than 29,000 women, there was limited power to detect associations due to the small number of women with pregnancies affected by birth defects in the study.

## Conclusions

In conclusion, we observed that optimal folic acid supplementation was associated with a decreased prevalence of birth defects, especially of the cardiovascular system and nervous system, in Shaanxi province, Northwestern China. These findings may have important public health implications for the prevention of birth defects in Northwest China. Further prospective cohort and RCT studies are needed to confirm the preventive effects of folic acid supplementation.

## Methods

### Study design and participants

Data were collected from a large population-based survey conducted between August and November 2013 in Shaanxi province, Northwest China. The participants were women who were pregnant between August 2011 and August 2013 and gave birth at a gestational age of ≥28 weeks. A sample size of 30,000 was estimated assuming a prevalence of birth defects of 1.4%, a relative error of 15%, a design effect of 2.0, two-tailed α = 0.05, and an expected response rate of 80%. Considering the imbalanced population distribution and fertility level between rural and urban areas in the province, 10 urban districts and 20 rural counties were randomly selected, and a stratified multistage sampling method was employed to obtain the sample. First, six townships were randomly sampled from the chosen counties, and three streets were randomly sampled from the chosen districts. Second, six villages were randomly sampled from a chosen township, and six communities were randomly sampled from the chosen streets. Third, 30 participants were randomly sampled from the chosen villages, and 60 participants were randomly sampled from the chosen communities. Approximately 32,400 participants were expected to be sampled in the study. A total of 30,027 women were eventually enrolled in the survey (response rate: 92.68%). We excluded 823 women with unclear pregnancy outcomes or missing covariates, leaving a total of 29,204 women in this study. Written informed consent was obtained from each participant at the start of the survey.

### Data collection

In the survey, all questionnaires, including the birth defects questionnaire and family questionnaire, and the field survey program were designed by Xi’an Jiaotong University Health Science Center. Face-to-face field surveys and data collection were carried out by uniformly trained field staff from Xi’an Jiaotong University Health Science Center. All the information was collected at the local village clinics and community health service centers. Our work was supported by local hospitals and health administration departments and the Ministry of Health in Shaanxi province.

A team of experts on the diagnosis of birth defects was established for this project. This team was composed of several senior medical technicians from the obstetrics and gynecology, ultrasound and pathology departments of the First Affiliated Hospital of Xi’an Jiaotong University. All cases of birth defects were diagnosed by the expert team using the ICD-10 to consistently and accurately code the diagnoses. For children with external anomalies, we collected their medical records and photographed the malformations for the final diagnosis. For children with congenital internal malformations, such as cardiovascular anomalies, we collected their medical records and conducted a free ultrasound examination at the First Affiliated Hospital of Xi’an Jiaotong University for the final diagnosis.

The family questionnaire was designed to collect information on maternal sociodemographic characteristics (including age, ethnicity, education, marital status, address of residence, occupation) and maternal exposure to periconceptional risk (including lifestyle, illnesses, periconceptional stress, exposure to harmful factors in the periconceptional environment, family history of diseases and periconceptional folic acid supplementation).

### Classification of birth defects

Birth defects were classified according to the International Classification of Diseases Clinical Modification Codes, tenth edition, (ICD-10) as congenital malformations, deformations, and chromosomal abnormalities (Codes Q00–Q99), which include nervous system defects (Q00–Q07); eye, ear, face and neck defects (Q10–Q18); cardiovascular system defects (Q20–Q28); respiratory system defects (Q30–Q34); oral clefts (Q35–Q37); digestive system defects (Q38–Q45); defects of the genital organs (Q50–Q56); urinary system defects (Q60–Q64); musculoskeletal system defects (Q65–Q79); other defects (Q80–Q89); and chromosomal abnormalities (Q90–Q99).

### Definitions of main variables

The information on folic acid supplementation and covariates was collected from the participants through face-to-face interviews conducted by well-trained interviewers via standard family questionnaires. The folic acid supplementation information included dosage per day, timing of use, and the period of regular use before and during pregnancy. This information was further confirmed by family members.

Optimal folic acid supplementation was defined regularly taking folic acid only or multiple micronutrients (≥400 µg folic acid per day) from 3 months before pregnancy through the first trimester of pregnancy for a duration of more than 3 consecutive months. Nonoptimal folic acid supplementation was defined as nonfolic acid supplementation, folic acid supplementation during the second and third trimesters of pregnancy, or folic acid supplementation for less than 3 months between the 3 months before through the 3 months after conception.

Other important covariates were defined. Infectious disease was defined as febrile episodes (>38 °C), influenza/common cold (influenza-like symptoms, seasonal influenza), infections of the kidney, bladder, urinary tract and genital tract and other infections affecting multiple organ systems (e.g., HIV infection, chicken pox) during pregnancy. Tobacco exposure was defined as active smoking (≥one cigarette per week for 3 consecutive months during pregnancy) or passive smoking (≥15 minutes of smoke inhalation per day for 1 consecutive month during pregnancy). Environmental risk factor exposure was defined as living within one kilometer of a coal mine, paper factory, cement factory, or power plant during pregnancy.

### Quality control

All of the interviewers were trained with the same guidelines before the study. A quality control system was constructed during the study, and the interviewers checked themselves and were checked by other interviewers and by a supervisor. When errors and missing values were detected, participants were reinterviewed immediately.

### Statistical analysis

We categorized 29,204 participants into optimal folic acid supplementation and nonoptimal folic acid supplementation groups. Of the 29,204 participants, 10,593 (36.27% of 29,204) had optimal folic acid supplementation. The PS was a function of multiple covariables and represents the combined action of multiple covariates^29^. Given the imbalance of baseline characteristics between the two groups, PS matching was used; PS matching can balance potential confounders between two groups with a similar randomized treatment and can reduce selection bias^8–10^. Then, we estimated the PS for each participant using a multivariable logistic regression model^29^, in which the level of folic acid supplementation was modeled using all the baseline participant characteristics shown in Table 2. We used the nearest neighbor within the caliper to match each participant with optimal folic acid supplementation with a participant with nonoptimal folic acid supplementation; thus, 9,293 (87.73% of the 10,593) participants with optimal folic acid supplementation were matched to 9,293 participants with nonoptimal folic acid supplementation and a similar estimated PS^31^. In our matching algorithm, we matched each participant with optimal folic acid supplementation with a participant with nonoptimal folic acid supplementation who had a similar PS to five decimal places. The nearest neighbor within caliper matching function was as follows:

$${\rm{C}}({P}_{i})=\mathop{\min }\limits_{j}||{P}_{i}-{P}_{j}||,j\in {I}_{0}$$

$$||{P}_{i}-{P}_{j}|| < \varepsilon ,j\in {I}_{0}$$

*P*_*i*_: propensity score of the optimal folic acid supplementation group; *P*_*j*_: propensity score of the nonoptimal folic acid supplementation group; *I*_*0:*_ the set of the nonoptimal folic acid supplementation group; C(*P*_*i*_): the matching nonoptimal folic acid supplementation participant for the optimal folic acid supplementation participant; ε: tolerance for matching (caliper). The absolute standardized difference based on the chi-square and Student’s t-tests was used to compare the baseline characteristics of the participants with optimal folic acid supplementation and those with nonoptimal folic acid supplementation before and after matching^10,32^. The prematch mean PS for the participants with optimal folic acid supplementation and nonoptimal folic acid supplementation were 0.39809 and 0.34259, respectively (absolute standardized difference = 70.58%, P < 0.001). After matching, the mean PS for the higher of the participants with optimal folic acid supplementation and nonoptimal folic acid supplementation were 0.38425 and 0.38425, respectively (absolute standardized difference = 0.01%, P = 0.999).

Then, we used univariate conditional logistic regression models to estimate the association of optimal folic acid supplementation with various birth defects and overall birth defects. We conducted sensitivity analyses using two approaches to assess the robustness of our findings regarding the effect of folic acid supplementation on birth outcomes to changes in the analytic approach. To address concerns about incomplete matching, we analyzed data from all 29,204 participants using GEE adjustment for PS and all baseline covariates and subclassification based on tertiles of PS.

The data were managed by a database using EpiData Version 3.02 (The EpiData Association, Odense, Denmark), and data entries were duplicated. All of the analyses were performed with STATA version 12.0 software (STATA Corporation, College Station, TX, USA). The level of significance was set at P < 0.05.

### Ethics statement

The Human Research Ethics Committee of the Xi’an Jiaotong University Health Science Center approved this study (No. 2012008). Written informed consent was obtained from all adult participants after a detailed explanation of the research. All research was performed in accordance with relevant guidelines and regulations.

## Data Availability

All data generated or analyzed during this study are included in this published article.
